# Hematological findings in COVID-19 and their correlation with severity of Disease

**DOI:** 10.12669/pjms.39.3.6836

**Published:** 2023

**Authors:** Namra Mahmood, Zahra Riaz, Arooj Sattar, Mehwish Kiran

**Affiliations:** 1 Namra Mahmood, MBBS, M.Phil. Assistant Professor, Pathology. Central Park Medical College, Lahore - Pakistan; 2 Zahra Riaz, MBBS, M.Phil. Senior Demonstrator, Department of Pathology. Central Park Medical College, Lahore - Pakistan; 3 Arooj Sattar, MBBS, M.Phil. Assistant Professor, Pathology. Central Park Medical College, Lahore - Pakistan; 4 Mehwish Kiran, Senior Registrar, Pulmonology. Central Park Medical College, Lahore - Pakistan

**Keywords:** COVID-19, Severity, Hematological parameters, Neutrophil lymphocyte ratio, Total leukocyte count, Total white cell count, Absolute neutrophil count, Lymphocyte count, Platelet count, PT, APTT, Length of hospital stay

## Abstract

**Objective:**

To evaluate the efficacy of hematological parameters to predict severity of COVID-19 patients.

**Method:**

This was a cross-sectional comparative study conducted at Central Park Teaching Hospital, Lahore in COVID ward and COVID ICU between April 23, 2021 to June 23, 2021. Patients of all ages and both genders with positive PCR admitted in the COVID ward and ICU during this time span of two months were included in the study. Data was collected retrospectively.

**Results:**

This study included 50 patients with male to female ratio of 1.38:1. Though males are more affected by COVID-19 but the difference is not statistically significant. The mean age of the study population was 56.21 and the patients in the severe disease group have higher age. It was observed that in severe/critical group the mean values of total leukocyte count 21.76×10^3^ μI (p-value= 0.002), absolute neutrophil count 71.37% (p-value=0.045), neutrophil lymphocyte ratio (NLR) 12.80 (p-value=0.00) and PT 11.9 seconds (p-value=0.034) and the difference was statistically significant. While in severe/critical group, the mean values of hemoglobin 12.03g/dl (p-value=0.075), lymphocyte count 28.41% (p-value=0.8), platelet count 226×10^3^ μI (p-value=0.67) and APTT 30.7 (p-value=0.081) and the difference was not significantly different between groups.

**Conclusion:**

It can be concluded from the study that total leucocyte count, absolute neutrophil count and neutrophil lymphocyte ratio can predict in-hospital mortality and morbidity in COVID-19 patients.

## INTRODUCTION

World Health Organization declared COVID-19 outbreak a pandemic which is caused by corona virus (SARS-CoV-2) family. This was first reported in Hubei province, Wuhan China at the end of 2019.^1^ Corona virus has single stranded RNA with lipoprotein envelope.^2^ It causes acute respiratory tract infection with variable level of severity. In addition to that, other body systems like cardiovascular, gastrointestinal and hemopoietic systems are also affected.^3^

Complete blood count is the most common first line investigation done in all the patients, which help and facilitate in diagnosis and to assess the response of treatment. Increased white blood count with neutrophilia and decrease in lymphocyte, eosinophil and basophil count are more pronounced in severe cases. In addition to that, D-dimer levels are also elevated.^4^

A review article from Sri Lanka showed lymphopenia and thrombocytopenia in patients with COVID-19. Lymphopenia is seen in about 69.62% whereas thrombocytopenia is seen in about 20-50% patients.^5^ In one of the studies conducted in Bangladesh, it was observed that D-dimers, C-reactive protein and ferritin are good indicators of disease severity in patients with COVID-19 infection.^1^ Similar observations were observed in a study conducted in Iran which showed higher D-dimer and deranged coagulation profile along with thrombocytopenia and lymphopenia.^6^

Ratio of white blood cells to neutrophils and platelet count can help to assess the severity of the disease with specificity and sensitivity, this observation was made in Sweden.^7^ Similar findings were observed by many scientists in local population that hemoglobin levels, total leukocyte count, differential leukocyte count and platelet count can help in predicting the severity of the disease and finding the outcome of the patients.^3^

The aim of the present study was to know the efficacy of hematological parameters in predicting mortality and morbidity in COVID-19 infection. Hematological parameters are first line investigation to be requested and available in almost every small and large healthcare setup. The findings of the study can be compared with local and international data, so the local guidelines can be established which will help in better management of COVID-19 patients and will result in reduction in the mortality and morbidity.

## METHODS

This was a cross-sectional comparative study conducted in the Central Park Teaching Hospital, Lahore in COVID ward and COVID ICU between April 23, 2021 to June 23, 2021. Ethical permission was taken from ethical committee and institutional research board. (Ref. CPMC/IRB-No/1313 on Feb 11, 2022.) Patients’ data was collected retrospectively on a pre-formed proforma containing patient information regarding demographic data, disease severity, disease outcome and haematological parameters.

### Inclusion & Exclusion Criteria:

- All patients admitted in the COVID ward and ICU during this time span of two months were included in the study.
- Patients of all ages and both genders admitted to the COVID ward and COVID ICU during this time frame with positive PCR for COVID-19 were included in the study.
- Patients under the age of 18 years, pregnant females, those with negative PCR and hematological malignancy were excluded from the study.

Demographic and clinical data was taken from the patient record files and laboratory data was obtained from hospital laboratory. Study population was divided into three groups; mild, moderate and severe/critical disease according to the WHO interim guidelines.^8^

All categorical data including gender, mortality and oxygen (O_2_) saturation and numerical data like hemoglobin, white blood cell count, neutrophil lymphocyte ratio, absolute neutrophil count, lymphocyte count, PT and APTT were entered and analyzed by using SPSS version 22. Continuous variables were presented by mean and standard deviation and were compared by using independent t-test and for categorical data chi-square test was used. A p-value of ≤0.05 was taken as significant.

## RESULTS

**Fig.1:**
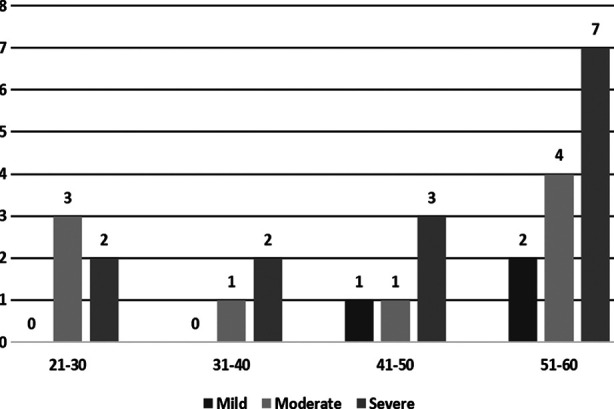
Age group distribution in different clinical severity groups.

This study included 50 patients of both genders, among these 29(58%) were males while 21(42%) were females with male to female ratio of 1.38:1. Though males were more affected by COVID-19 but the difference is not statistically significant. The mean age of the study population was 56.21 years. When age was analyzed between study subgroups, it was noted that patients in the severe disease group had higher age compared to mild and moderate disease groups. The median age of patients in different groups is shown in the Table-I. Severe disease group patients were of significantly higher age compared to others with p-value of 0.003. Age group distribution in different severity groups are shown in Fig.1.

**Table-I:** Basic characteristics of study population (n=50).

	Total	Mild	Moderate	Severe/Critical		p-value
Age (Median)		46	52	65	t-test	0.003
***Gender***						
Males	29(58%)	7	9	13	Chi-square	0.06
Females	21(42%)	1	7	13		
***O_2_ saturation***						
>93%	29(58%)	01	18	10	Chi-square	0.01
<93%	21(42%)	12	09	0		
Mortality	19(38%)	2	3	14	Chi-square	0.03

Patients with COVID-19 were assessed for their O_2_ saturation; 21 patients had O_2_ saturation below 93% and 29 had above 93%. When it was analyzed for severity groups, it was observed that more patients in severe/critical group had O_2_ saturation <93%. Difference between groups with regard to O_2_ saturation was significant with p-value of 0.01. In-hospital mortality was higher in severe/critical group and difference between groups was significant with p-value of 0.03. The basic features of the study population are shown in the Table-I.

Hematological parameters were compared between different clinical severity groups and findings are shown in the Table-II. It was observed that total leukocyte count (p-value=0.002), absolute neutrophil count (p-value=0.045), neutrophil lymphocyte ratio (p-value=0.00) and PT (pvalue=0.034) were significantly more in the severe/critical group. While Hemoglobin (pvalue=0.075), lymphocyte count (p-value=0.8), platelet count (p-value=0.67) and APTT (pvalue=0.081) were not significantly different between the groups. These findings are shown in the Table-II.

**Table-II:** Hematological parameters in different severity groups.

	Units	Mild Disease	Moderate disease	Severe Disease	t-test (p-value)
Hemoglobin	g/dl	11.23±2.88	12.68±2.13	12.03±2.88	0.075
Total leukocyte count	×10^3^/μl	21.76±8.07	17.42±7.84	21.76±9.07	0.002
Absolute neutrophil count	%	71.37±28.58	74.25±21.18	71.37±28.58	0.045
Lymphocyte count	%	22.50±28.41	20.06±20.84	28.41±28.41	0.8
Neutrophil lymphocyte ratio		11.38±11.05	8.20±8.20	12.80±10.56	0.000
Platelets	×10^3^/μl	246.25±56	271.06±79	226.91±86	0.67
PT	Seconds	10.7	10.9	11.9	0.034
APTT	Seconds	25.3	27.3	30.7	0.081

## DISCUSSION

It is observed in the present study that hematological parameters correlate with COVID-19 affected patients’ disease severity. Males were more affected than females in the current study. Mousavi et al. also showed male predominance, though difference was not statistically significant, as observed in the current study.^9^ Jin JM et al. also described similar findings in which he further elaborated that males had more severe disease compared to females.^10^

Patients with severe disease were of higher age compared to moderate and mild disease**.** The median age of the patients with severe disease was 65 years. Previous studies have also shown higher age in patients with severe disease and in non-survivors. Laninis et al. also reported significantly high age in mortality group.^11^ Another study by Pozdnyakova has shown median age in patients with COVID-19 admitted in ICU as 64.12 years which is similar to the present study.^12^

The present study demonstrated that white blood cell count, absolute neutrophil count and neutrophil lymphocyte ratio was significantly high in severe disease group compared to other groups. Previous studies have also shown that these parameters are significantly high in severe and critically ill patients with COVID-19. A study by Bellan et al compared these variable between those who were discharged and died. He reported significant difference between two groups with respect to white cell count, neutrophil count, neutrophil lymphocyte ratio, eosinophil and basophils.^13^ Similarly, Asghar et al. described significantly higher total leukocyte count and neutrophil lymphocyte ratio in patients with critical disease, and those who died compared to those patients who had mild disease.^14^ Neutrophil lymphocyte ratio is an independent predictor of prognosis in patients with COVID-19 infection.^14^,^15^ Another study conducted by Toori et al. also showed that higher neutrophil lymphocyte ratio is associated with more severe disease.^16^ The current study did not find any significant relation between lymphocytes count and disease severity, but one study in Pakistan showed significant relationship between lymphopenia and disease severity.^17^ Another study conducted in China has shown that NLR is associated with increased severity of the disease and also the mortality in patients with COVID-19 infection.^18^

In the current study, no correlation was found between severity of disease and hemoglobin level and platelet count. A study conducted by Taj et al. also described no relation between these parameters and clinical severity groups^3^ but another study conducted by Rehman T et al. showed significant difference with respect to hemoglobin and platelet count.^19^ Similarly, Elderdery AY et al. also reported significant difference of hemoglobin and platelet count between patients with COVID-19 and controls.^20^

In coagulation profile, PT and APTT was also analyzed between groups and only PT was found to be significantly different in patients with severe disease group. A study by Huang et al; Wan et al., Wu et al. and Zhou et al. reported raised PT in patients with severe disease and in non-survivors.^21^-^24^ Current study couldn’t find any significant difference with regard to APTT but a study conducted by Taj S et al. has shown significant difference in clinical severity groups.^3^

Mortality rate in the present study was 38% which was very high compared to the previous studies conducted locally. This may be due to the fact that majority of our patients were from severe/critical group who had high mortality and secondly it was done in a tertiary care hospital where mostly serious patients were admitted. Mortality rate of 5.8% was reported by Mehra et al. and another study by Taj S et al. reported mortality rate of 7.9%.^3^,^25^

### Limitation:

It was a single centre study where randomization was not done. Confounding factors cannot be excluded completely.

## CONCLUSION

It can be concluded from the study that total white cell count, absolute neutrophil count and neutrophil lymphocyte ratio can predict in-hospital mortality and morbidity in COVID-19 patients.

## RECOMMENDATION

At the time of admission, keen eye on basic laboratory tests can help in risk stratification of the patients. This will help in the management of the patients and will result in reduction in mortality and morbidity.

### Authors’ Contributions:

**NM:** Study design, data collection, writing the manuscript, formulation of tables reviewed and approved. She is also responsible for the integrity and accuracy of the study. **ZR:** Statistical analysis, interpretation of data, manuscript writing and revising it critically for important intellectual content. **AS:** Statistical analysis, interpretation of results, reviewed and approved the manuscript. **MK:** Data collection, writing the manuscript, formulation of tables, reviewed and approved.
